# A metagenomic study of the gut microbiome in Behcet’s disease

**DOI:** 10.1186/s40168-018-0520-6

**Published:** 2018-08-04

**Authors:** Zi Ye, Ni Zhang, Chunyan Wu, Xinyuan Zhang, Qingfeng Wang, Xinyue Huang, Liping Du, Qingfeng Cao, Jihong Tang, Chunjiang Zhou, Shengping Hou, Yue He, Qian Xu, Xiao Xiong, Aize Kijlstra, Nan Qin, Peizeng Yang

**Affiliations:** 1 The First Affiliated Hospital of Chongqing Medical University, Chongqing Key Lab of Ophthalmology, Chongqing Eye Institute, Chongqing, 400016 China; 2 Realbio Genomics Institute, Shanghai, 201114 China; 3 Beijing Institute of Ophthalmology, Beijing Tongren Eye Center, Beijing Tongren Hospital, Capital Medical University, Beijing Ophthalmology & Visual Sciences Key Lab, Beijing, 100730 China; 4 Shenzhen Jinrui Biotechnology, Co. Ltd., Shenzhen, 518000 China; 5 University Eye Clinic Maastricht, Maastricht, The Netherlands; 6 Shanghai Tenth People’s Hospital Affiliated to Tongji University, Shanghai, 200072 China

**Keywords:** Behcet’s disease, Gut microbiome, Metagenomic analysis, Fecal microbiota transplant

## Abstract

**Background:**

Behcet’s disease (BD) is a recalcitrant, multisystemic inflammatory disease that can lead to irreversible blindness. Microbial agents have been considered to contribute to the pathogenesis of this disease, but the underlying mechanisms remain unclear. In this study, we investigated the association of gut microbiome composition with BD as well as its possible roles in the development of this disease.

**Methods:**

Fecal and saliva samples were collected from 32 active BD patients and 74 healthy controls. DNA extracted from fecal samples was subjected to metagenomic analysis, whereas DNA extracted from saliva samples was subjected to 16S rRNA gene sequencing analysis. The results were used to compare the composition and biological function of the microbiome between patients and healthy controls. Lastly, transplantation of pooled fecal samples from active BD patients into B10RIII mice undergoing experimental autoimmune uveitis (EAU) was performed to determine the causal relationship between the gut microbiome and BD.

**Results:**

Fecal samples from active BD patients were shown to be enriched in *Bilophila* spp*.*, a sulfate-reducing bacteria (SRB) and several opportunistic pathogens (e.g., *Parabacteroides* spp*.* and *Paraprevotella* spp*.*) along with a lower level of butyrate-producing bacteria (BPB) *Clostridium* spp*.* and methanogens (*Methanoculleus* spp. *Methanomethylophilus* spp.). Analysis of microbial functions revealed that capsular polysaccharide transport system, oxidation-reduction process, type III, and type IV secretion systems were also increased in active BD patients. Network analysis showed that the BD-enriched SRB and opportunistic pathogens were positively correlated with each other, but they were negatively associated with the BPB and methanogens. Animal experiments revealed that fecal microbiota transplantation with feces from BD patients significantly exacerbated EAU activity and increased the production of inflammatory cytokines including IL-17 and IFN-γ.

**Conclusions:**

Our findings revealed that BD is associated with considerable gut microbiome changes, which is corroborated by a mouse study of fecal microbiota transplants. A model explaining the association of the gut microbiome composition with BD pathogenesis is proposed.

**Electronic supplementary material:**

The online version of this article (10.1186/s40168-018-0520-6) contains supplementary material, which is available to authorized users.

## Background

Behcet’s disease (BD) is a chronic, multisystemic, inflammatory disorder characterized by recurrent attacks of oral ulcers, genital ulcers, skin lesions, and uveitis [57, 65]. BD is a great public health concern, as it is one of the most common causes of blindness. Studies have shown that dysregulated autoimmunity associated with genetic factors and infectious agents may contribute to its pathogenesis [57, 65]. Aberrant activities of T helper 1 (Th1), Th17, and regulatory T (Treg) cells were observed in BD patients [57, 65]. Recent studies showed that the gut microbiome might play a crucial role in modulating Th1, Th17, and Treg cells [1, 22, 58]. Dysbiosis of the gut microbiome has been observed in many inflammatory autoimmune diseases, including multiple sclerosis (MS) [3], rheumatoid arthritis (RA) [68], ankylosing spondylitis [62], systemic lupus erythematous (SLE) [19], and inflammatory bowel disease (IBD) [38]. Likewise, alteration of the gut microbiome was also shown to be associated with BD [9, 52, 54]. However, these studies on BD were based on 16S rRNA gene amplicon sequencing method that has its limitations, including potential skewing the microbial composition due to amplification bias [45] and inability to examine most microbes at species and strain level [24]. To thoroughly investigate the microbial components associated with the disease, we herein performed a metagenomic study of the gut microbiome in BD patients along with a group of healthy controls. Our results show that BD patients display a characteristic composition and functional profile of the gut microbiome different from that of healthy controls. Importantly, fecal transplantation into B10RIII mice undergoing experimental autoimmune uveitis (EAU) with pooled stool samples from BD patients exacerbated the eye symptoms in these animals. Based on the findings of this study, we propose a model to explain the association between the gut microbiome composition and BD pathogenesis.

## Methods

### Study participants

Thirty-two ocular BD patients receiving no medication except topical corticosteroids and cycloplegics for at least 1 month along with 74 sex-, age-, and BMI-matched healthy controls were recruited for this study. Individuals with cardiovascular diseases, diabetes, other inflammatory diseases, and infectious diseases were excluded. Diagnosis of BD was based on the diagnostic criteria of the international study group for BD [26]. Prior to sample collection, the subjects had not received antibiotics or probiotics for at least 1 month. Active BD was defined according to the presence of active intraocular inflammation. The demographic data (e.g., age, gender, and BMI), clinical manifestations and treatment information of all patients and healthy controls were recorded (Additional file 1: Table S1). The study was approved by the Ethics Committee of Chongqing Medical University. Signed informed consent was obtained from all participants at the beginning of the study. All procedures were performed in accordance with the Declaration of Helsinki.

### Sample collection and DNA extraction

Fresh fecal samples and saliva samples were collected from the recruited subjects or B10RIII mice (fecal samples only) and were transported to our laboratory with an ice pack within 2 h. All samples were then frozen immediately and stored at − 80 °C prior to analyses.

DNA was extracted from the samples using the QIAamp Fast DNA Stool Mini Kit (Qiagen, Hilden, Germany) according to the manufacturer’s instructions. Briefly, 20 μl proteinase K solution (20 mg/ml) and 100 mg zirconium beads (0.1 mm) were added to the pellet before the mixture was fully homogenized on a Mini-Beadbeater (FastPrep, Thermo Electron Corporation, USA) and then supplemented with buffer AL. The resulting mixture was incubated at 70 °C for 10 min and supplemented with 200 μl ethanol (96%) before being loaded onto the QIAamp Mini spin column and centrifuged at 8000 g for 1 min. The column was washed successively with 500 μl buffer AW1 and 500 μl buffer AW2. Finally, DNA was eluted with 100 μl buffer AE. DNA concentration was measured using a NanoDrop (Thermofisher, USA). The integrity and size of the extracted DNA were examined with electrophoresis on 1% agarose gel containing 0.5 μg/ml ethidium bromide.

### Sequencing of 16S rRNA gene amplicon

DNA from 58 saliva samples (15 from untreated active BD patients and 43 from healthy controls) was subjected to amplification of polymerase chain reaction (PCR) using primers directed at hypervariable region 3–4 (V3–V4) of the 16S rRNA gene (341F and 806R). The PCR products were quantified using Qubit (Invitrogen, Carlsbad, CA, USA), multiplexed at even concentration and subject to 300 bp pair-end sequencing on Illumina MiSeq platform (Illumina, Inc., San Diego, CA, USA). The resulting raw reads were filtered using a quality control criterion (i.e., minimum average quality score = 20; maximum number of ambiguous N base = 3) and matched to sequences spanning the entire V3–V4 amplicon using PANDAseq [36]. A total of 2,642,548 merged sequences were generated from all samples, resulting in an average yield of 45,561 ± 6485 sequences/sample. Merged sequences with 97% nucleotide sequence identity (97% identity) were binned into operational taxonomic units (OTUs) using UPARSE [11]. Based on the RDP classifier, a representative sequence of each OTU was assigned to a taxonomic level in the RDP database using 0.8 as the minimum confidence threshold [8]. The OTU table was rarefied to 29,723 sequences/sample. Alpha and beta diversity were calculated using QIIME with the default parameters [25].

### Metagenomic sequencing

Following the Illumina TruSeq DNA Sample Prep v2 Guide (Illumina, Inc., San Diego, CA, USA), we constructed the DNA paired-end libraries with an insert size of 500 bp for the 76 fecal samples (24 from untreated active BD patients and 52 from normal controls). The quality of all libraries was evaluated using an Agilent bioanalyzer (Agilent Technologies, Wokingham, UK) and the DNA LabChip 1000 kit. All samples were subject to 150 bp paired-end sequencing on an Illumina HiSeq 4000 platform (Illumina, Inc., San Diego, CA, USA).

Raw reads were filtered to trim nucleotides from the 3′ end using a quality threshold of 30 and remove adaptor contamination and low-quality reads (e.g., reads containing more than 50% nucleotides below Q30, reads short than 70 bp, and reads mapped to the human genome based on alignment with SOAPaligner 2.21 [28]). As a consequence, an average of 95.82% high quality reads was obtained from all samples.

### De novo assembly and construction of the gene catalog

To construct the gut gene catalog, SOAPdenovo [33] (version 2.04) was used to assemble the high quality reads from each sample into contigs. MetaGeneMark [41] (version 3.26) was used to predict open reading frames (ORFs) in contigs. To obtain a non-redundant gene set, pairwise comparison of predicted ORFs (filtered with a length of 100 bp) was performed using CD-HIT [29] (version 4.5.7) at 95% identity and 90% coverage. The final non-redundant gene catalog contained 1,502,032 microbial genes, which had an average length of 766 bp. Functional annotations were carried out by BLASTP search against the eggNOG 3.0 database [21] and KEGG database [23] (*e* value ≤ 1e − 5 and high-scoring segment pair scoring > 60). For each functional feature (KO in KEGG database or OG in eggNOG), we estimated its abundance by accumulating the relative abundance of all genes belonging to this feature.

### Taxonomic and gene profiling

SOAPalign2.21 was used to align clean reads to the microbial reference genomes downloaded from the National Center for Biological Information (NCBI, http://www.ncbi.nlm.nih.gov) (74,201 genomes, including 65,770 bacterial, 898 archaeal, 1508 fungal and 6025 viral genomes). The taxonomic relative abundance profile was generated following the procedure described by Qin et al. [48]. Reads aligned to multiple taxa were allocated proportionally to read counts uniquely mapped to these taxa (normalized by genome length). The same strategy was performed for the gene abundance profile.

### MGS analysis

Genes were clustered into Metagenomic Species (MGS) following the method described by Le Chatelier et al. [27] and Nielsen et al. [40], as described in the following section. First, based on the abundance variation across all individuals, the differentially abundant genes (*P* < 0.05 in Wilcoxon test) with a Spearman correlation coefficient (rho) > 0.8 were clustered using single-linkage clustering according to their abundance variation across all individuals. Second, the mean abundance of each cluster with more than 25 genes was computed, and clusters with rho > 0.8 were fused to produce MGS. The taxonomic annotation and abundance profile of each MGS were generated according to the taxonomy and the relative abundance of the corresponding genes. Following the previously described procedure [48], each MGS was assigned to a taxonomical assignment from strain to super kingdom level, with a threshold of > 90% of the genes in this MGS as well as with the best hit to the same phylogenetic group (> 95% identity and > 90% overlap of query).

### Classifier

We used random forest model (randomForest package in R) to build the classifier based on the abundance profiling of MGSs. The predictive performance (estimated by 10-fold cross-validation) was optimal when the top 13 most discriminatory MGSs were included, showing that these 13 MGS had the most discriminatory power. Using these 13 MGSs as predictors, a mean classification error of 0.18 was achieved, and the area under the receiver operating characteristic (ROC) curve (AUC) was 0.811(95% confidence interval (CI) 0.70–0.92, sensitivity 87.5%, and specificity 67.3%). The ROC curve was plotted using the pROC R package.

### Correlation network

SparCC [15] was used to construct the association network of BD. Briefly, bootstrapping of 100 repetitions was used to compute the *P* value for each correlation. The resulting two-sided pseudo *P* values were adjusted for multiple comparisons using the Benjamini and Hochberg correction [2]. Only significant correlations with an adjusted *P* < 0.1 were presented in the network, which was visualized with Cytoscape3.0.2.

### Animals and reagents

B10RIII mice were purchased from Jackson Laboratory (Bar Harbor, ME, USA) and housed under standard (specific pathogen free) conditions. Human interphotoreceptor retinoid binding protein peptide spanning amino acid residues 161–180 (IRBP161–180, SGIPYIISYLHPGNTILHVD) was purchased from Shanghai Sangon Biological Engineering Technology and Services Ltd. Co. Complete Freund’s adjuvant (CFA) supplemented with 1.0 mg/ml *Mycobacterium tuberculosis* strain (MTB) was purchased from Sigma-Aldrich (St. Louis, MO, USA). The animal study was approved by the Ethics Committee of the First Affiliated Hospital of Chongqing Medical University and was carried out according to the ARVO Statement for the Use of Animals in Ophthalmic and Vision Research.

### Fecal microbiome transplantation in mice

Eleven B10RIII mice were first treated with an antibiotic cocktail containing ampicillin (1 mg/ml), neomycin (1 mg/ml), metronidazole (1 mg/ml), and vancomycin (0.5 mg/ml) (all purchased from Sigma-Aldrich) for 3 weeks as described previously [37, 49]. Stool samples from five untreated BD patients and five healthy controls were used to colonize the B10RIII mice. The metadata of these donors are shown in Additional file 1: Table S1. Fecal microbiota transplant was performed as described previously [69]. Briefly, a fecal sample from each donor was resuspended in PBS to a final concentration of 200 mg/ml and then equal volumes of donor suspensions were pooled. Each mouse was orally administered with 200 μl of the pooled fecal suspension once a day for 1 week after the antibiotic treatment (four mice receiving pooled fecal suspension from untreated active BD patients, four mice receiving pooled fecal suspension from healthy controls, and the others transferred with PBS only). At day 7, mice were sacrificed and cecal content was obtained for subsequent analysis.

### EAU induction and lymphocyte response evaluation

Mice were immunized subcutaneously at the base of the tail and bilateral thighs with 25 μg human interphotoreceptor retinoid-binding protein peptide spanning amino acid residues 161–180 (IRBP_161–180_, SGIPYIISYLHPGNTILHVD) peptide in 100 ml PBS, emulsified 1:1 *v*/*v* in complete Freund’s adjuvant (CFA), supplemented with 1.0 mg/ml *M. tuberculosis* (MTB) after fecal transplantation. Clinical and histological scoring was performed as described previously [6, 59]. The spleen was removed from immunized mice on day 14, and spleen cells were separated. RNA was extracted from spleen cells and used as the template to generate cDNA using the PrimeScript RT reagent Kit with gDNA Eraser (TaKaRa, Otsu, Japan). Expression of IL-17 and IFN-γ mRNA was determined by real-time PCR analysis using the Power SYBR Green PCR Master MIX (Biosystems, Warrington, UK) on an ABI 7500 Real-time PCR System (Applied Biosystems, CA, USA). The primers for IL-17, IFN-γ, and β-actin were as follows: IL-17 forward: 5′-CTCAACCGTTCCACGTCACCCT-3′ and reverse: 5′-CCAGCTTTCCCTCCGCATT-3′; IFN-γ forward: 5′-TCAAGTGGCATAGATGTGGAAGAA-3′ and reverse: 5′-TGGCTCTGCAGGATTTTCATG-3′; β-actin forward: 5′-GGCTGTATTCCCCTCCATCG-3′ and reverse: 5′-CCAGTTGGTAACAATGCCATGT-3′.

### Statistical analyses

The non-parametric Wilcoxon test (wilcox.test in R) was employed to analyze the statistical significance of the gene, KO, OG, enzyme, and different taxonomic (phylum, genus, species) levels. The relative abundance of these features was subjected to statistical analyses. The Benjamini-Hochberg method was used for correction in multiple comparisons in which a *P* value < 0.1 was considered significant. Enriched features with an adjusted *P* < 0.1 were identified, and the enrichment group was then determined according to a higher rank-sum value. Linear discriminant analysis (LDA) effect size (LEfSe) analysis [51] was used to determine the features (organisms, KOs, or OGs) most likely to explain differences between the BD and healthy control group. Different features with an LDA score cut-off of 2.0 were identified. KO modules with a reporter score of *Z* > 1.6 was identified as an enriched module, which was computed from the *Z* scores of individual KOs [14, 43].

## Results

### Altered gut microbial composition in BD patients.

**Fig. 1:**
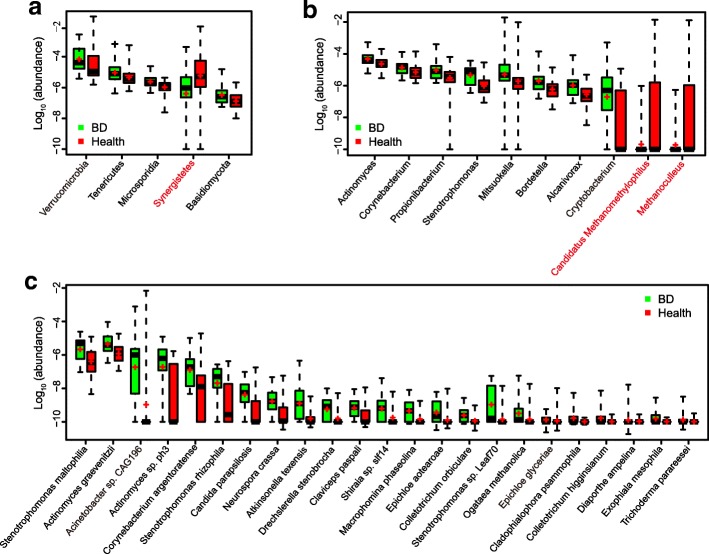
Phyla (**a**), genera (**b**), and species (**c**) showing significant differences in fecal metagenome profiles when comparing BD patients with healthy controls. Differentially abundant phylotypes were identified by Wilcoxon rank sum test (FDR < 0.1, corrected by the Benjamini and Hochberg for multiple comparisons). Only the top 5 phyla and top 10 genera are shown. The phylotypes enriched in healthy group are colored red. The relative abundance was shown by boxplot. Boxes represent the inter quartile ranges, lines inside the boxes denote medians, and ‘+’ denotes means

To investigate the composition and function of the gut microbiome in BD patients, we sequenced the metagenome of 76 fecal samples (24 from untreated active BD patients and 52 gender- and age-matched healthy controls) (Additional file 1: Table S1). An average of 6.4 ± 2.1 Gb clean reads was generated per sample (Additional file 1: Table S2). The clean reads were aligned to the reference genomes from the National Center for Biological Information (NCBI) and Human Microbiome Project (HMP) catalogs. The results showed that *Bacteroidetes*, *Firmicutes*, and *Proteobacteria* were dominant phyla in both BD patients and healthy controls. The relative abundances of 97 genera and 23 species were significantly different between the BD patients and healthy controls (Additional file 1: Table S3). Up to 96 genera were enriched in BD patients, including 38 belonging to the fungal phylum *Ascomycota*, followed by two bacterial taxa, *Proteobacteria* (15 genera), and *Actinobacteria* (15 genera). Five genera were enriched in healthy controls and 2 of them were methanogens (*Methanoculleus* and *Methanomethylophilus*). At the species level, 23 microbes were enriched in the BD patients. Some of these are considered opportunistic pathogens, such as *Stenotrophomonas* spp*.*, *Actinomyces* spp*.*, and *Corynebacterium* spp*.* (Fig. 1). Hence, our analysis revealed that BD patients displayed apparent differences in their gut microbial composition. Interestingly, at both genus and species levels, fungi showed a significant presence (49 of the 96 differential genera and 16 of the 23 differential species, Additional file 1: Table S3 and Fig. 1c).

**Fig. 2:**
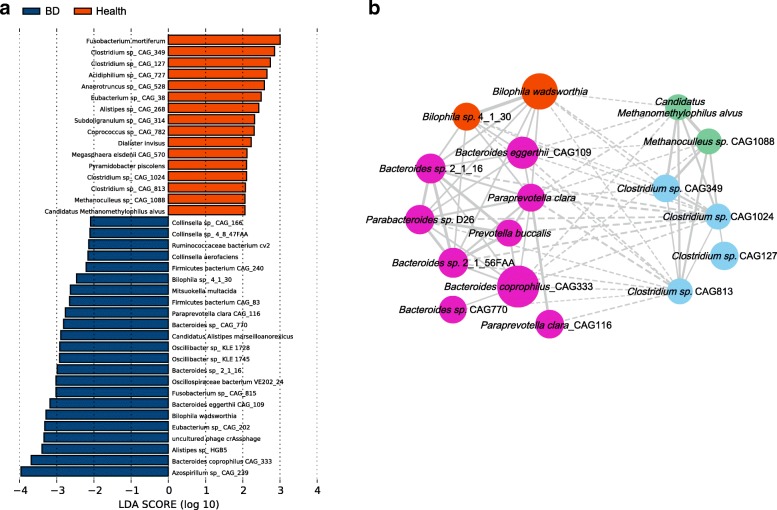
Linear discrimination analysis (LDA) effect size (LEfSe) analysis results comparing the BD and healthy groups. **a** Histogram of the LDA scores computed for genera differentially abundant between BD subjects and healthy controls. The LDA scores (log10) > 2 are listed. **b** SparCC network plot of co-abundance and co-exclusion correlations between differentially abundant SRB, BPB, methanogens, and opportunistic pathogens. Each node represents one species, and two nodes are linked if the correlation was significant (two-sided pseudo *p* ≤ 0.1 based on bootstrapping of 100 repetitions). Lines between nodes show positive correlations (solid lines) or negative correlations (dashed lines). The node size is proportional to the mean relative abundance of species in the enriched population. Nodes were colored as follows: orange, sulfate-reducing bacteria; purple, lactate-producing bacteria; blue, butyrate-producing bacteria; green, methanogens

To further investigate the features more likely to explain the differences between the BD and healthy groups, linear discriminant analysis (LDA) effect size (LEfSe) was performed by coupling standard tests for statistical significance with additional analyses examining biological consistency and effect relevance. Features with an LDA score cut-off of 2.0 were identified as being different. The results showed that sulfate-reducing bacteria (SRB) *Bilophila* spp*.* and opportunistic pathogens including *Parabacteroides* spp*.*, *Paraprevotella* spp*.*, and *Fusobacterium* spp*.* were significantly enriched in BD, whereas methanogens *Methanoculleus* spp., *Methanomethylophilus* spp., and butyrate-producing bacteria (BPB) *Clostridium* spp*.* were negatively associated with the patients (Fig. 2a). These results together disclosed that SRB and opportunistic pathogens were enriched in BD patients while methanogens and BPB were enriched in healthy controls.

### Metagenomic species (MGSs) profiling for the BD patients

**Fig. 3:**
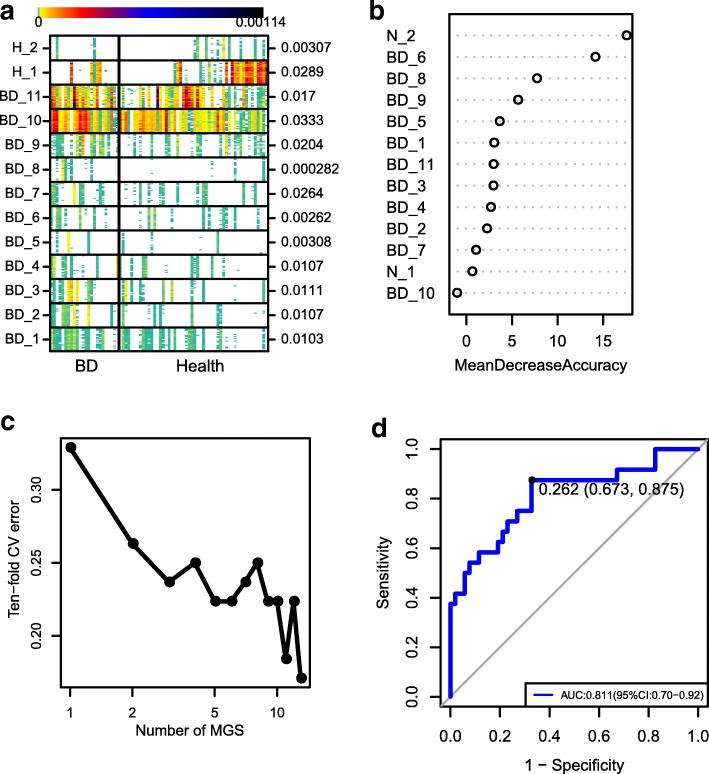
MGSs analysis. **a** The heatmap of 25 ‘tracer’ genes abundance for each MGS were shown. Individuals are represented along the horizontal axis. Abundance of genes in rows is indicated by color gradient (white, not detected), and the enrichment significance is shown on the right with *P* value by Wilcoxon test. **b** The relative importance of each MGS in the predictive random forest model using the mean decreasing accuracy. **c** Relationship between the numbers of MGSs included in random forest model and the corresponding predictive performance (estimated by 10-fold cross-validation). **d** The ROC curve for the random forest model using 13 MGSs

MGS profiling was also performed to investigate the BD-associated microbial components. A total of 17,896 microbial genes were found to be significantly different between the BD patients and healthy controls. Subsequent relative abundance profiling identified 13 MGSs. Of those, 11 were enriched in the BD group and 2 were enriched in the healthy control group (Fig. 3a). Twelve of the MGSs could be assigned to a known microbial taxon (Additional file 1: Table S4). We noticed that the genus *Bilophila*, *Alistipes*, *Paraprevotella*, and species *Faecalibacterium prausnitzii* were BD-enriched MGSs and that the strain *Clostridium sp. CAG:127* was an enriched MGS in the healthy controls (Additional file 1: Table S4). Of these MGSs, one BD-enriched MGS belonged to SRB *(Bilophila)*, one belonged to opportunistic pathogens (*Paraprevotella*), whereas one BD-depleted MGS belonged to BPB *(Clostridium sp. CAG:127)*. Overall, the results of MGS profiling (Fig. 3) were in agreement with that of the microbial composition analysis (Figs. 1 and 2). Using the 13 MGSs as microbiome markers to discriminate the BD group from the healthy control group, the area under the receiver operating characteristic curve (AUC) was 81.1%, and the 95% confidence interval (CI) was 70–92% (Fig. 3d).

### Examination of interactions among differentially abundant microbes

SparCC network plot of co-abundance and co-exclusion correlations [15] was constructed to investigate the possible interactions between the differentially abundant microbes. Two patterns were apparent from the network. First, SRB and opportunistic pathogens formed one cluster whereas BPB and methanogens formed the other. Intra-cluster associations were mostly positive whereas inter-cluster associations were mostly negative (Fig. 2b). These results indicated that there were complex interactions between these microbes, although more studies are needed to further elucidate these interactions.

### Altered gut microbial function in BD patients

A LEfSe analysis was performed to investigate whether the differences in gut microbiome composition also had functional consequences concerning the expression of certain genes. We identified 25 KEGG (Kyoto Encyclopedia of Genes and Genomes database) orthologues (KO) and 13 eggNOG (evolutionary genealogy of genes: Non-supervised Orthologous Group database) orthologues (OG) that were significantly different between the BD patients and healthy controls (Additional file 1: Tables S5 and S6). The results showed that several KOs or OGs associated with the oxidation-reduction process were enhanced in BD patients. Furthermore, one BD-depleted KO was found to be associated with lysozyme, which has antibacterial properties due to the fact that it degrades peptidoglycans in the bacterial cell wall [12]. At the module level, the BD group was enriched in the capsular polysaccharide transport system, as well as in the types III and IV secretion systems (Additional file 1: Table S7).

### Altered oral microbial composition in BD patients

Recurrent oral ulcers are one of the most common clinical features in BD. To understand the profile of the oral microbiome and its association with the gut microbiome in BD, 16S rRNA gene amplicon sequencing was performed for 58 saliva samples (15 from untreated active BD patients and 43 from gender- and age-matched healthy controls). The results showed that *Bifidobacteriales* were enriched in BD at the order level. At the genus level, *Bifidobacterium*, *Prevotella*, and *Scardovia* were enriched in BD (Additional file 1: Table S8). Similar results were obtained with LEfSe analysis (Additional file 2: Figure S2). These results suggested that BD patients also have alterations in their oral microbiota composition.

### Effect of fecal transplantation on experimental autoimmune uveitis (EAU) in B10RIII mice

The aforementioned experiments showed that the composition and function of the gut microbiome is different in active BD patients when compared to healthy controls. We further investigated whether the feces from active BD patients could influence the development and severity of uveitis in an experimental animal model (EAU). The EAU model in mice is currently the best model for human autoimmune uveitis including BD and is widely used to study the pathogenetic mechanisms, prevention, or treatment strategies [7]. Stool samples from five BD patients or healthy controls were used to colonize B10RIII mice pretreated with antibiotics. Based on analyses of the Shannon index and PCoA, the recipient mice, with either the BD patients or healthy controls as the feces donor, showed significantly higher microbial diversity than, and different microbial functions from, their non-recipient counterparts (Additional file 2: Figure S1). Importantly, we observed an enrichment in *Bilophila*, *Alistipes*, and *Paraprevotella* in the BD-treated group, which was in line with the MGS results of the human gut microbiome (Additional file 2: Figure S1c, Additional file 1: Table S9).

**Fig. 4:**
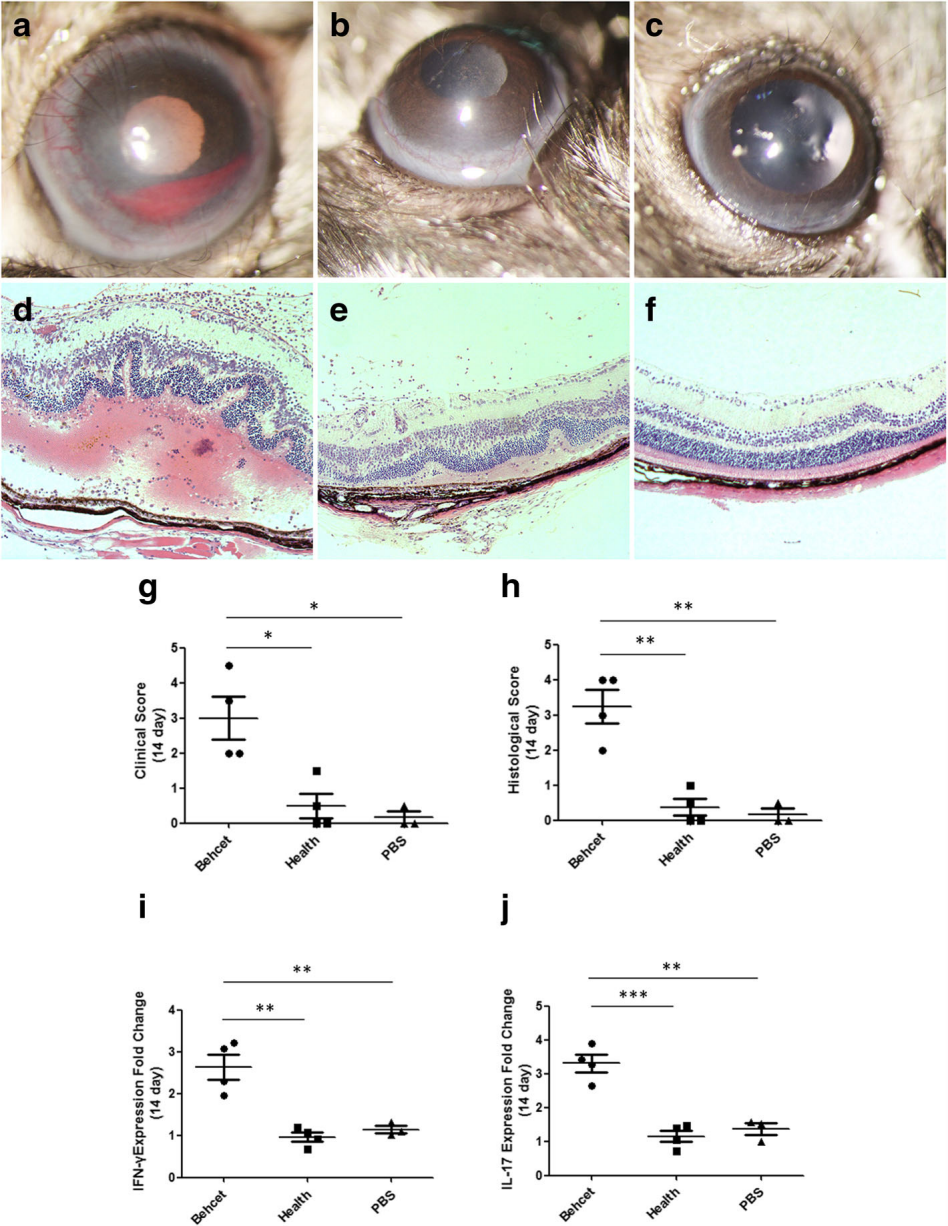
Effect of transplantation of feces from BD patients on EAU. Pooled feces from active BD patients transferred to B10RIII mice by oral gavage. Pooled feces from healthy individuals and PBS were transferred to mice as control groups. EAU was induced by immunization with IRBP_161–180_. On day 14 after EAU induction, clinical and histological scores of BD patients’ feces-treated group (**a** and **d**), healthy’ feces-treated group (**b** and **e**), and PBS-treated group (**c** and **f**) were determined. Combined data in (**g**) for clinical score and (**h**) for histological score. Each point represents an individual eye. The horizontal bars denote the average scores of each group. The spleens were also removed from EAU mice on day 14 after induction. IFN-γ (**i**) and IL-17 (**j**) mRNA levels were evaluated by real-time PCR

After fecal transplantation, mice were immunized with the retinal protein peptide IRBP_161–180_ combined with CFA for EAU induction. At 14 days after immunization, we compared the clinical and histological scores as well as the expression of BD-associated cytokines in spleens between the BD-recipient group and healthy control-recipient group. The results showed that severe uveitis manifested by ciliary injection, corneal edema, and cells in the aqueous humor as well as posterior synechiae were observed in the BD-recipient group, which was supported by the clinical scores ranging from 2 to 4.5 (Fig. 4a). In the healthy control-recipient group and PBS-treated group, only a slight intraocular inflammatory reaction including conjunctival and/or ciliary injection was observed, evidenced by the clinical scores ranging between 0 and 1.5 (Fig. 4b, c). The BD-recipient mice showed a more severe clinical manifestation than the healthy control-recipient group and PBS-treated group (both *P* = 0.012, Fig. 4g). Histological analysis showed that the retinal architecture exhibited severe folding in the BD-recipient group (Fig. 4d). Massive infiltrates of inflammatory cells were observed throughout the retina and choroid as well as in the vitreous cavity in the group of mice receiving fecal transplants from BD patients (Fig. 4d). In comparison, only scattered inflammatory cells were observed in the vitreous cavity of mice receiving feces from healthy controls as well as in the PBS-treated animals. No significant folding of the retinal architecture was observed in these two groups (Fig. 4e ,f). In line with our clinical results, the histological score in the BD-recipient group was significantly higher than that in the healthy control-recipient group and PBS treated-group (both *P* = 0.002, Fig. 4h). The expression of IL-17 and IFN-γ mRNA was significantly increased in the BD-recipient group as compared with the healthy control-recipient group (IL-17, *P* < 0.001; IFN-γ, *P* = 0.009; Fig. 4i, j) or PBS-treated group (IL-17, *P* = 0.006; IFN-γ, *P* = 0.014; Fig. 4i, j).

## Discussion

In this study, we show that patients with BD have a distinct gut microbiome signature. BD is a systemic vascular disorder influenced by genetic and environmental factors [9, 57, 65]. As a host factor, the gut microbiome has recently received considerable attention for its potential role in the pathogenesis of BD [9, 52, 54], although a causal relationship has yet to be established. We applied the metagenomic method to characterize the disease-associated microbiome, whereby we identified possible gut microbiome biomarkers (including microbial composition and function) in BD patients. Fecal transplantation into mice using BD patients’ feces exacerbated the development of intraocular inflammation as well as IL-17 and IFN-γ production in these animals. Taken together, these results indicate that gut microbiome composition might contribute to the development of this disease.

We established a gut microbial gene repertoire of BD and found that some opportunistic pathogens and SRB were enriched in the BD patients, while BPB and methanogens were enriched in the healthy controls. These data are in agreement with two recent studies from Italy and Japan [9, 54] that suggested that gut microbiome abnormalities are implicated in BD pathogenesis.

SRB are pro-inflammatory bacteria and have been shown to be involved in a number of inflammatory or immune diseases, including type 2 diabetes (T2D) [47], metabolic syndrome [66], and inflammatory bowel disease (IBD) [46]. *Bilophila wadsworthia*, a species of SRB, promotes a Th1-mediated immune response in dietary-fat-induced colitis [10]. SRB species inhibit butyrate β-oxidation and degrade butyrate [39, 46]. Butyrate is a beneficial metabolite that protects the integrity of the intestinal epithelial barrier and maintains host immune homeostasis by inducing differentiation of Treg cells [16, 60]. A decreased butyrate level leads to intestinal epithelium barrier dysfunction and facilitates the expression of various inflammatory components such as the microbe-associated molecular pattern (MAMP) or pathogen-associated molecular pattern (PAMP) factors, which can affect intestinal epithelial cells (IEC) [44]. Moreover, hydrogen sulfide (H_2_S), which is a cytotoxic byproduct of SRB and exerts pro-inflammatory effects at high concentrations [4, 55], can exacerbate intestinal epithelial barrier damage.

BPB are considered a group of beneficial bacteria and can ferment dietary fibers to produce butyrate [42]. As such, they have a protective role in human colonic health [32] and may have a therapeutic effect in IBD [17]. Previous studies have shown that the level of BPB was decreased in IBD patients [17, 61] and T2D patients [47], a finding that is in agreement with our current observation in BD patients. Methanogens are another group of bacteria revealed in this study to be associated with BD. Methane, which is generated by methanogens in the gastrointestinal tract of humans, is believed to ameliorate oxidative stress injury and can suppress the inflammatory response in various tissues and organs including the retina [63], colon [67], liver [64], and brain [53]. The present study also showed that depletion of methanogens in BD is associated with enriched functional components related to the oxidation-reduction processes. Interestingly, co-occurrence network analysis showed that SRB were negatively associated with BPB and methanogens in the BD group. A high level of H_2_S and a low level of butyrate were detected when SRB and BPB were co-cultured with lactate-producing bacteria [35]. Because SRB can use lactate and hydrogen as substrates to produce H_2_S, SRB are in competition for substrates with lactate-utilizing bacteria and hydrogen-consuming bacteria such as BPB and methanogens. This might explain the negative correlation between SRB and BPB or methanogens found in the BD patients. The interactions between these three groups of bacteria have not yet been fully examined in humans. We believe that the metagenomic data obtained in our study may help studies on other autoimmune or metabolic diseases involving these bacteria such as RA, IBD, and MS [3, 38, 68]. The hypothesis mentioned above is, of course, speculative and further research is needed to support it.

Several opportunistic pathogens such as *Stenotrophomonas* spp., *Actinomyces* spp*.*, *Corynebacterium* spp., and *Paraprevotella* spp*.* were observed to be enriched in active BD patients and were implicated in various diseases in susceptible hosts [5, 34, 56]. *Stenotrophomanas maltophilia*, which was found to be enriched in BD patients, is associated with the type IV secretion system (T4SS) [50]. Interestingly, T4SS, along with type III secretion system (T3SS), was also found herein to coincide with BD-enriched functional components (Additional file 1: Table S7). Opportunistic pathogens may need T3SS or T4SS to deliver effector proteins into host cells, thereby inducing inflammatory responses [18, 20]. On the other hand, these opportunistic pathogens can produce lipopolysaccharide (LPS) and peptidoglycans (PGN), which function as so-called MAMPs or PAMPs and can trigger an inflammatory response via host cell receptors such as TLR4 and TLR2 [30]. The PGN-related functional component was also shown to be enriched in the BD patients investigated (Additional file 1: Table S6). These findings are consistent with previous studies from our group in which we showed that PGN/TLR2 and LPS/TLR4 pathways are involved in the aberrant immune response observed in BD [13, 30, 31]. We also demonstrated that TLR2 gene polymorphisms were involved in the susceptibility to BD and that the TLR2 mRNA expression was increased following stimulation with PGN in healthy carriers of the susceptibility genes of BD [13].

**Fig. 5:**
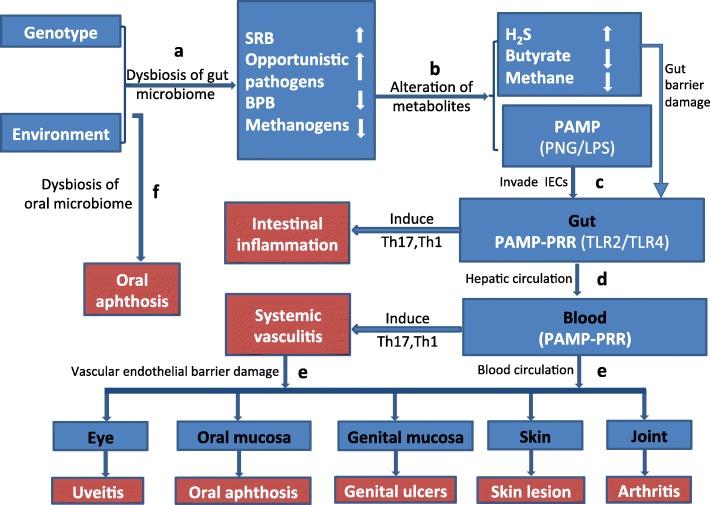
Chart of possible mechanisms explaining the relation between gut microbiome composition and development of Behcet’s disease. **a** Dysbiosis of the gut microbiome might be caused via dietary intake in individuals carrying the susceptibility genes for BD. The dysbiosis of the gut microbiome in BD is characterized by enriched sulfate-reducing bacteria (SRB) and some opportunistic pathogens in association with depleted butyrate-producing bacteria (BPB) and methanogens. **b** Gut metabolism in BD shows an overwhelming presence of H_2_S and shortage of butyrate and methane. This abnormal environment can contribute to the intestinal epithelial barrier damage and facilitate effector molecules or pathogen-associated molecular pattern (PAMP) to invade the intestinal epithelial cells (IEC). **c** The PAMPs including PNG/LPS combine with their corresponding pattern recognition receptors (PRR) TLR2/TLR4 on IEC. This process leads to chronic inflammation involving hyperactivation of T helper 1 (TH1) and T helper 17 (TH17) cells in the gut. **d** The effector molecules or PAMP migrate to blood vessels through the hepatic circulation. Then, they recognize the receptors of TLR/TLR4 on vascular endothelial cells (VEC) and induce systemic vasculitis via the subsequent activation of TH1 and TH17 cells. **e** A damaged vascular endothelial barrier due to the systemic vasculitis. The effector molecules or PAMP can further migrate to organs or tissues such as the eye, joint, skin, oral, and genital mucosa though the damaged vascular endothelial barrier. Subsequently, the PAMP recognize the receptors TLR2/TLR4 in these organs or tissues, which result in various clinical manifestations of BD, such as uveitis, arthritis, skin lesions, oral, or genital ulcers. **f** Since oral ulcers (aphthosis) can be induced by both disturbances of the oral microbiome and dysbiosis of the gut microbiome, it presents as the most common clinical feature in BD

Based on the aforementioned findings, we propose herein a hypothesis to explain the association between the gut microbiome signature and BD. BPB and methanogens are beneficial bacteria that can maintain proper host immune homeostasis. However, the balance may be tipped by an overgrowth of some opportunistic pathogens such as SRB, *Stenotrophomonas* spp., *Actinomyces spp.*, and *Paraprevotella* spp*.*, which leads to a decrease of BPB and methanogens. These abnormalities can induce intestinal epithelial barrier damage and facilitate the effector molecules or MAMP/PAMPs (PGN/LPS) to enter the IEC. Meanwhile, the process induces an overexpression of corresponding PRRs (TLR2/TLR4). Subsequently, as reported in previous studies, the ligands PGN/LPS can interact with receptors such as TLR2/TLR4. A decreased level of BPB and methanogens may lead to an impaired control of the PGN/LPS- induced TLR-MyD88 pathway, which in turn induces a series of inflammatory reactions including systemic vasculitis in BD (for more details see Fig. 5 and Table 1). Of note, in this hypothesis, we would like to emphasize that it is the “disease-associated microbial community” (DAMC) rather than a single pathogen that plays a crucial role in disease pathogenesis. In this study, SRB and some other opportunistic pathogens together constitute the DAMC of BD.

**Table 1:** Links between the disturbed gut microbiome and their possible immune signaling pathway in BD patients

Disturbed gut microbiome	Enriched group	Species	Possible PAMP	Possible PRP	Possible related immune cells	Possible related immune cytokine	Reference
SRB	BD	*Bilophila* spp.	LPS, H2S	TLR4	Upregulation Th1 cells	IFN-**γ**	[7, 39, 60]
Opportunistic pathogens	BD	*Stenotrophomonas* spp., *Actinomyces* spp.,*Corynebacterium* spp., *Paraprevotella* spp.	T3SS, T4SS, LPS, PGN	TLR2, TLR4	Upregulation Th1 and Th17 cells	Unknown	[5, 18, 34, 35, 50, 56]
BPB	N	*Clostridium* spp.	Butyrate	TLR-MyD88	Unregulation Treg cell	IL-10	[16, 46, 66]
Methanogens	N	*Methanoculleus* spp., *Methanomethylophilus* spp.	Methane	TLR-MyD88	Macrophages	IL-10	[32]

*SRB* sulfate-reducing bacteria; *BPB* butyrate-producing bacteria; *PAMP* pathogen-associated molecular pattern; *PRP* pattern recognition receptors; *BD* Behcet’s disease; *N* normal controls; *PGN* peptidoglycan; *LPS* lipopolysaccharides; *TLR* Toll-like receptors; *H2S* hydrogen sulfide; *T3SS* type III secretion system; *T4SS* type IV secretion system

Examination of the oral microbiome composition of BD patients revealed an abnormal level of *Prevotella*, which were also observed in the gut. This implies that changes in the bacterial composition can play a role in disease pathogenesis via both the intestines as well as the oral cavity. Our results are in part similar to a recent study on the oral microbiome in BD from the USA [52], which showed an increase of *Bifidobacterium* and a decrease of *Neisseria*.

We also attempted to identify bacterial biomarkers that may help in the diagnosis BD by generating 13 different MGS markers. When using these markers, we were able to distinguish BD patients from healthy controls with a moderate degree of certainty (AUC = 82.69). Analysis of a large size of samples is needed and further study should be carried out to explore whether these biomarkers can be useful in prevention, diagnosis, or prognosis of BD.

To determine whether the gut microbiome contributes to the development of BD, fecal transplantation was performed in mice undergoing autoimmune uveitis. We demonstrated that mice, colonized with whole gut microbiome from BD patients, showed an exacerbation of disease activity and an excessive production of pro-inflammatory cytokines. These results might confirm the hypothesis that the “DAMC” serves as a pathogen-like entity and contributes to the development of intraocular inflammatory disease. We must point out, however, that the gut microbiome in mice colonized with patients’ feces was not in complete conformity with the MGS results in patients. This might be due to the influence of the indigenous microbial community in mice, since the antibiotics treatment was stopped when the fecal transplantation was performed. This issue may be addressed by using germ-free mice in future experiments.

Taken together, we found a disturbed composition of the gut microbiome in BD patients and analyzed possible mechanisms underlying its role in disease pathogenesis. Based on the results, we propose a model of DAMC to explain the microbial involvement in the pathogenesis of BD. We realize that our model is speculative and hope that it will lead to further studies to elucidate the complex interactions between the gut microbiome and pathogenesis of this debilitating disease.

## Additional files

Additional file 1: Supplementary Tables. (XLSX 279 kb)

Additional file 2: Supplementary Figures. (PDF 975 kb)

## Abbreviations

AUC: Area under the receiver operating characteristic curve
BD: Behcet’s disease
BPB: Butyrate-producing bacteria
CI: Confidence interval
DAMC: Disease-associated microbial community
DAMP: Damage-associated molecular patterns
EAU: Experimental autoimmune uveitis
eggNOG: Evolutionary genealogy of genes: Non-supervised Orthologous Groups database
H_2_S: Hydrogen sulfide
HMP: Human Microbiome Project
IBD: Inflammatory bowel disease
IEC: Intestinal epithelial cells
KEGG: Kyoto Encyclopedia of Genes and Genomes database
KO: KEGG orthologues
LEfSe: Linear discriminant analysis effect size
LPS: Lipopolysaccharide
MAMP: Microbe-associated molecular pattern
MGSs: Metagenomic species
MS: Multiple sclerosis
NCBI: National Center for Biological Information
OG: eggNOG orthologues
ORFs: Non-redundant open reading frames
OTUs: Operational taxonomic units
PAMP: Pathogen-associated molecular pattern
PGN: Peptidoglycans
RA: Rheumatoid arthritis
SLE: Systemic lupus erythematosus
SRB: Sulfate-reducing bacteria
T3SS: Type III secretion system
Th cell: T helper cell
TLRs: Toll-like receptors
Treg cell: Regulatory T cell
