# Supplementary material for: A metagenomic study of the gut microbiome in Behcet’s disease
Source: Microbiome. 2018 Aug 4;6:135. doi: 10.1186/s40168-018-0520-6 (PMC6091101; doi:10.1186/s40168-018-0520-6)
Supplement: Supplementary file 2 — Supplementary Figures. (PDF 975 kb) [file 40168_2018_520_MOESM2_ESM.pdf]

Supplementary Figures

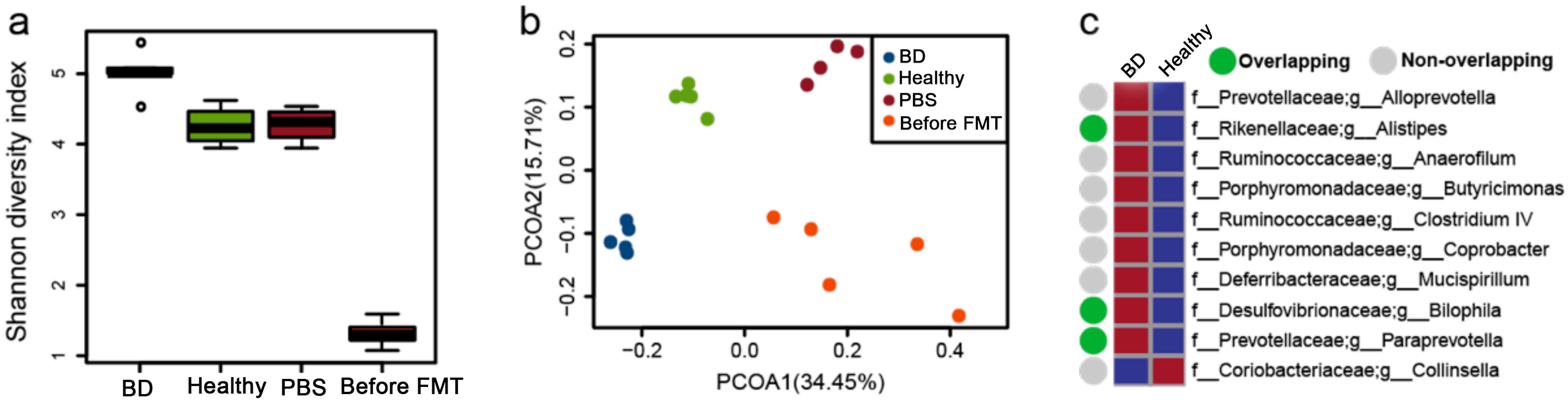

Figure S1. Post-transplant intestinal microbial profiles of mice. (a) Shannon index of mice at the genus level demonstrates significantly increased α diversity after (pooled) feces transferral from active BD patients and healthy individuals and PBS treatment. Boxes represent the inter quartile ranges, lines inside the boxes denote medians, and circles are outliers. (b) PCoA plots based unweighted unifrac distance of genera showed the different microbiota among BD patients' feces treated group, healthy' feces treated group, PBS treated group and pre-transplanted group. (c) Heat map comparing the abundance of altered genera between BD patients' feces treated group, healthy' feces treated mice. Red, more abundant; blue, less abundant (Wilcoxon rank sum test, adjusted *P* values <0.1, corrected by the Benjamini and Hochberg). Genera present consistent trend with the metagenomic analysis are marked with

green points, while inconsistent with gray points.

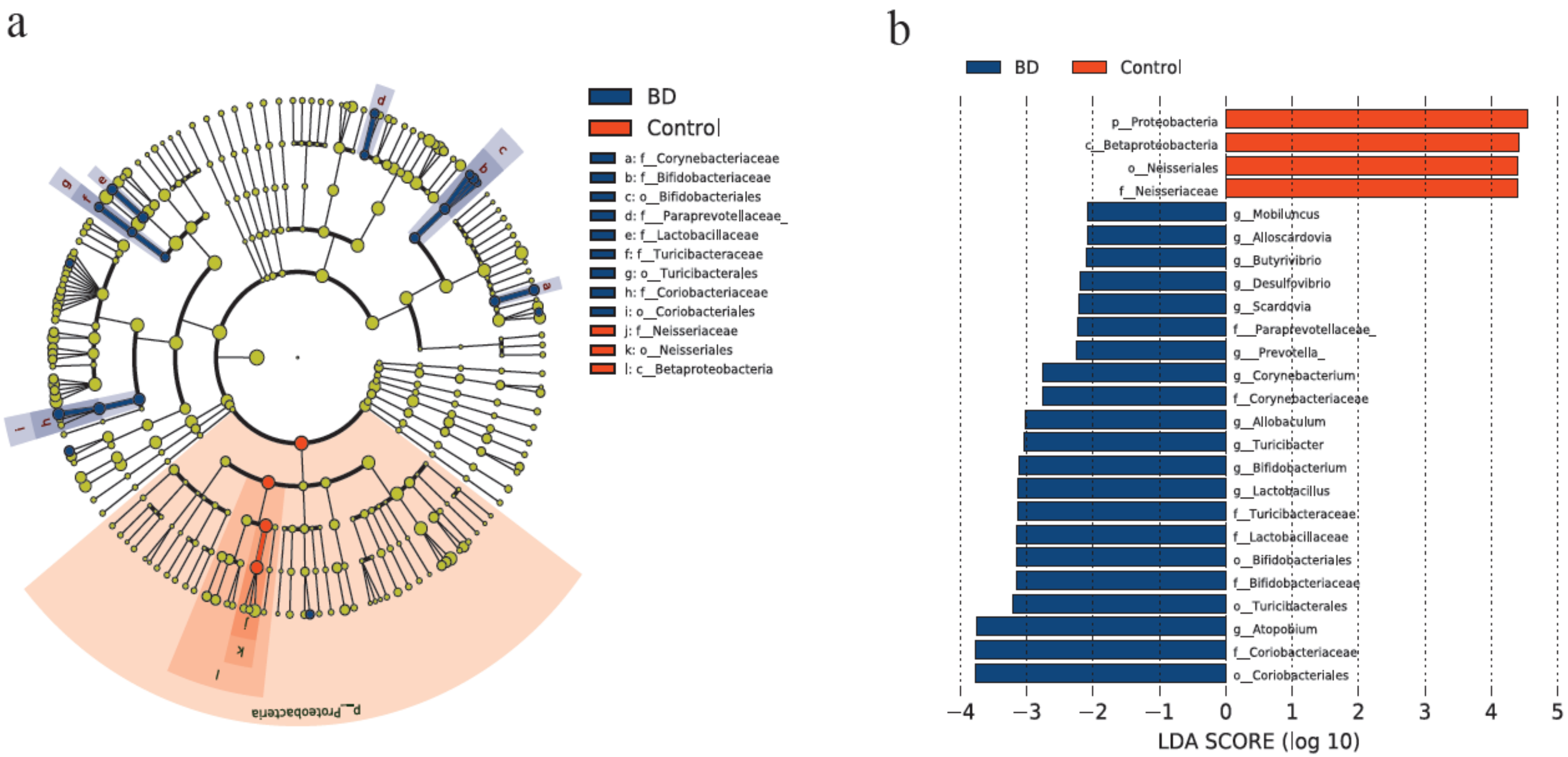

Figure S2. LEfSe analysis results comparing the controls and BD group using the 16s rRNA sequencing data of salivary samples.

(a) Cladogram showing the most discriminative bacterial clades identified by LEfSe. Colored regions/branches indicate differences in the bacterial population structure between BD subjects and healthy controls. Regions in red indicate clades that were enriched in controls, while regions in blue indicate clades that were enriched in BD subjects. (**b**) Histogram of the LDA scores computed for features differentially abundant between BD subjects and healthy controls.
